# Shielding effect of the smoke plume by the ablation of excimer lasers

**DOI:** 10.1186/s12886-018-0942-8

**Published:** 2018-10-23

**Authors:** Csaba Szekrényesi, Huba Kiss, Tamás Filkorn, Zoltán Zsolt Nagy

**Affiliations:** 1 Faculty of Health Sciences, Semmelweis University, Vas u. 17, Budapest, 1088 Hungary; 2 Department of Ophthalmology, Semmelweis University, Budapest, Hungary

**Keywords:** Excimer lasers, PMMA, Laser ablation, Refractive surgery

## Abstract

**Background:**

Shielding and scattering effect of the smoke plume column ejected from the laser ablated material is a well-known phenomenon. Debris evacuation system of the excimer laser equipment removes these particles, but insufficient air flow can result in undesired refractive outcomes of the treatment. The aim of this study was to reveal the effect of the air flow speed on the actual ablation depth.

**Methods:**

SCWIND AMARIS 500E flying spot excimer laser was tested in this study. A 150 μm phototherapeutic keratectomy (PTK) profile with 8 mm diameter was applied to the surface of polymethyl methacrylate (PMMA) plates. The velocity of the air flow was changed with adjustable air aspiration system. Ablation depth was measured with highly-precise contact micrometer.

**Results:**

The prediction model was statistically significant, F(1,8) = 552.85, *p* < 0.001, and accounted for approximately 98.7% of variance of ablation (*R*^*2*^ = 0.987, *R*^*2*^_adj_ = 0.986). Lower air flow speed resulted in a weaker ablation capability of the excimer laser.

**Conclusion:**

Air flow generated by the aspiration equipment is a key factor for the predictable outcomes of refractive treatment. Therefore, manufacturer inbuilt debris removal system should be regularly checked and maintained to ensure proper clinical and predictable refractive results.

## Background

Excimer laser devices are used worldwide for reshaping of the cornea and to change the refractive power of the corneal surface to achieve emmetropia. The high-precision, 193 nm laser pulses created by the excimer laser absorbed by the surface of the cornea and ablated a part of the corneal tissue with a 1 μm precision. The effects of the environmental factors regarding the fluence and efficacy of the excimer laser pulses are an investigated and deeply-researched area, and both of them is important and might have a long-term effect on the results of the excimer laser procedure [1–4]. Other general factors as temperature and humidity in the operation theater have to be kept in a given range. They might have an influence on the stable function and preset parameters of the optical system of the excimer laser and on the energy settings as well. The smoke plume generated during by the excimer laser pulses is a well-known phenomenon [5–7]. Beer-Lambert Law shows a non-linear relationship between the laser fluence (F) and depth of the ablation (d).

$$ \mathrm{d}=1/\upalpha \ast \ln \left(\mathrm{F}/{\mathrm{F}}_{\mathrm{th}}\right) $$

Where F_th_ is the ablation threshold and α is the ablation coefficient. These last two parameters depend on the material to be ablated [8]. The laser energy is absorbed by the material, surface molecules are photoablated and particles are ejaculated from the surface. The generated smoke plume shields and scatters the next laser pulses, decreases the energy load and the achieved ablated depth as well. Therefore to reach the planned ablation depth and profile, by the design of the excimer lasers the smoke plume evacuation system or debris module is an essential part of the device.

To the precise performance of the excimer lasers, PMMA (polymethyl methacrylate) plates are used for the calibration procedure and for regular maintenance [9]. Test treatments are applied to the surface of the PMMA plates. The ablated area is measured with contact or non-contact methods [10–15].

The study presented by Dorronsoro et al. [16] shows that the shielding and scattering effect produced by the smoke plume column has a significant influence to the ablation depth by shielding the laser fluence. In this experimental setting the ablation depth on PMMA plates were measured with 4 different air flow settings. Significant relationship was found by the speed of the air flow. From these three air speed settings a mathematical model was created, which suggests that the air flow speed should depends on the repetition rate.

In the present study the ablation depth was investigated during precise experimental conditions and by 10 different air flow speed settings and a statistical relationship was found between the air flow speed caused by the smoke plume evacuation system. Compared to the results of Dorronsoro in this article authors evaluated more technical settings of the smoke plume evacuation unit in order to reveal more accurate statistical relationship and clinical significance to achieve precise ablation depth and the best postoperative refraction.

## Methods

Last-generation SCHWIND AMARIS 500E flying spot excimer platform (SCHWIND eye-tech solutions Gmbh, Kleinostheim, Germany) was used in this study. This laser platform is able to perform myopic, hyperopic, astigmatic and higher order customized treatments as well. Relevant parameters are: 193 nm wavelength, 0.54 mm Super-Gaussian laser-spot profile, 500 Hz repetition rate, flying-spot ablation strategy.

The air flow speed was measured with TESTO 405-V1 anemometer (accuracy: ± (0.3 m/s ± 5% of mv)).

**Fig. 1:**
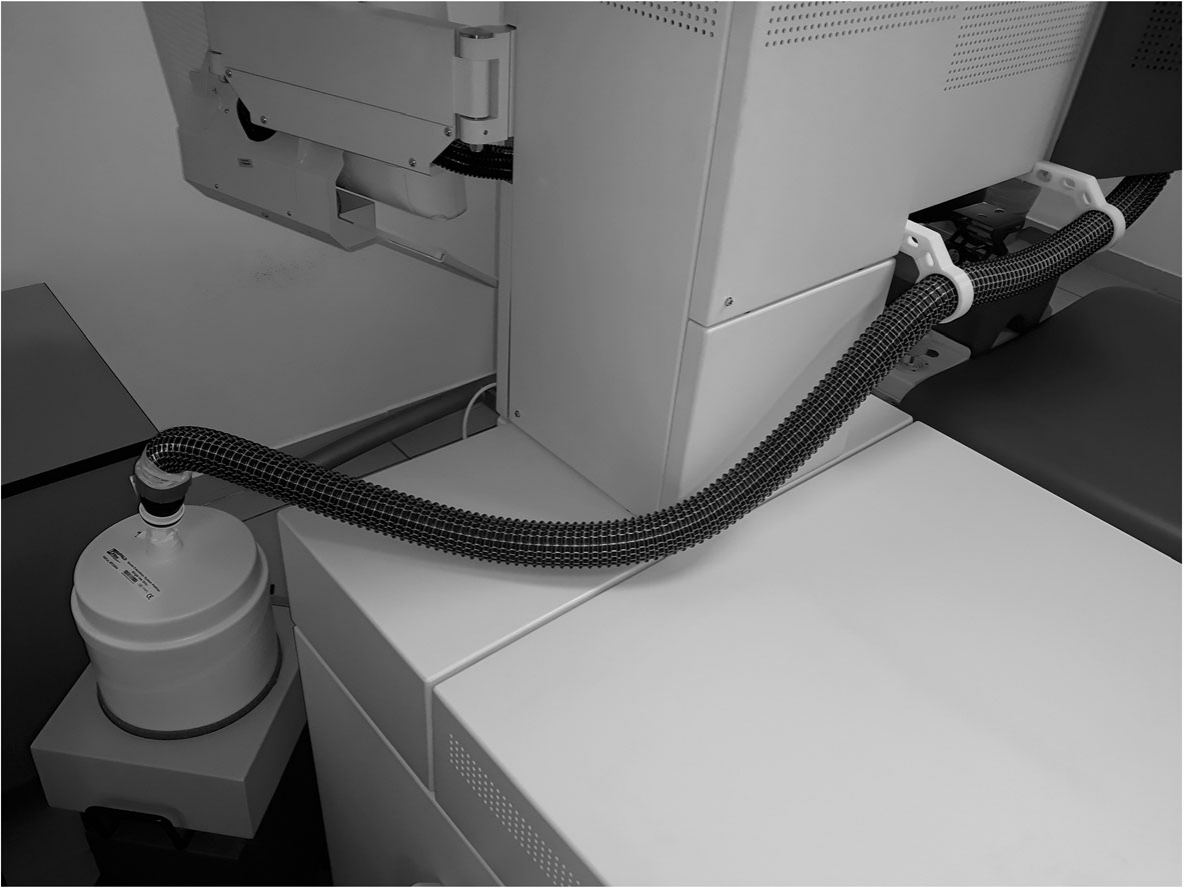
Photo about the experimental layout of the study

The SCHWIND AMARIS 500E used to these study works with a manufacturer-modified plume evacuator system, which based on the suction only. The fix speed, external plume evacuator offered by the manufacturer was exchanged in this study to adjustable external smoke evacuation equipment (Smoke Evacuator) (Edge Systems Corporations, Redondo Beach, CA, USA). Only the evacuator equipment was exchanged. The patient-side mechanics, the debris suction module, the debris nozzle, the air-pipelines and the main tube which leads to the external evacuator were identical with the manufacturer-offered plume evacuation system in all cases during the study. The experimental layout can be seen on the Fig. 1.

The voltage and current of the rotor of the evacuation system were adjusted manually with a button on the control panel of the equipment continuously from 1 to 9 scaled linearly. The evacuator system is able to work standalone, without the control of the laser device as well. The laser starts and stops the external evacuator, but it is possible to start and stop manually. Every suction speed measurement was conducted with the anemometer at the mouth of the debris suction nozzle in the same position.

Airflow speed was measured after the evacuator was set to a certain step. 150 μm PTK treatments were performed in 8 mm diameter on three PMMA plates without changing the setting of the evacuator. After finishing three treatments on the same level of suction speed, the airflow was measured again to find out, if the airflow speed was changed during the treatments. This procedure was repeated on 9 different settings of the evacuator from the lowest possible airflow to the maximum suction level, the air speed values were in the range of 4.6 to 7.4 m/s.

According to our experiences the suction speed was unchanged before and after the measurement of a certain setting of the evacuator. This indicates a steady, constant relation between the setting of the evacuator and the suction power. Since the suction characteristic of the evacuation system was unknown, the measured air velocity values were used for the statistical calculations. Once the evacuator was adjusted to a certain airflow setting, three ablations on three different PMMA plates were performed without any adjustment of the evacuator. Therefore the error of the evacuator was taken in the statistical calculation as a constant value, regarding to our airflow speed measurements before and after the ablation. Therefore the study did not calculate with the error propagation.

This external evacuation system is equipped with HEPA (high-efficiency particulate arrestance) pre- and main filter combination.

Round shaped flat PMMA plates (28 mm diameter, 4 mm height) were used for the measurement of the ablation depth of the test treatment. This plate is equal to the PMMA plate used by daily calibration procedure of the Wavelight Allegretto 400 (Alcon Wavelight, Erlangen, Germany) provided by the company. Energy calibration was performed and flatness of each PMMA plate was checked before the ablation with zeroing of the micrometer. Manufacturer-offered mechanical adapter holding a spirit level was applied to ensure the central position and the tilting of the surface of the samples to ensure the exactly perpendicular position of the optical axis of the instrument. Tilting of the adapter was corrected with thin metal sheets. The height and the horizontal adjustment were maintained during the whole measurement procedure. The PMMA plates are transparent, an artificial pupil was used by the backside surface of the PMMA made from a paper sheet with a round-shaped 4 mm diameter hole, to direct the position of the test treatment within the center of the round-shaped surface.

Humidity was during the measurement 35% ± 5%, temperature was 22.5 °C ± 0.5 °C in the operation theatre.

The achieved central ablation depth was measured with a calibrated contact micrometer, Inductive Dial Comparator 2000 (Mahr, Göttingen, Germany) with 901 R type standard contact point (accuracy: ±0.2 μm). The contact point of the micrometer is a ruby ball with 3 mm diameter, which measures in the center of the ablation area. The measurement area considered to be point-like. During this study every PMMA plate was measured once. Sixty seconds waiting time has been kept after the ablation and before the measurement to stabilize the surface of the PMMA.

The percent differences between each two ablation depths of the same air-flow velocity were calculated. A collapsed variable from the three measurements were calculated. Linear regression was conducted to predict the ablation depth from the air flow velocity. Statistical analysis was performed using OriginPro 9 (OriginLab Corp., Northampton, MA, USA). A *p*-value of **<** 0.05 was considered statistically significant.

## Results

**Fig. 2:**
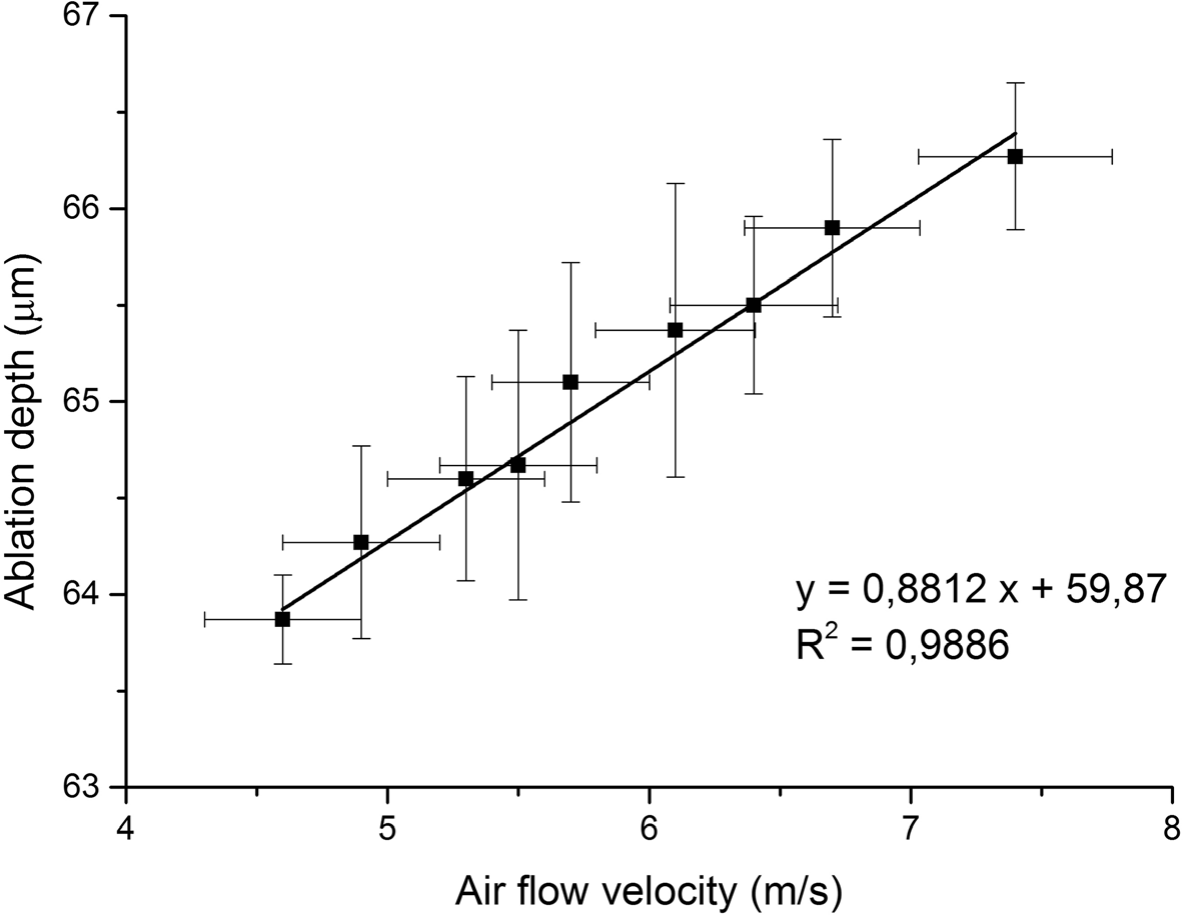
Correlation between the velocity of air flow and the ablation depth

As no significant difference was observed between the percent difference of every two measurements (percent differences: 0.00–2.29, M = 1.02, SD = 0.57) a collapsed variable was calculated from the three measurements, which is a mean value (Table 1). A simple linear regression was calculated to predict the central ablation depth based on the air velocity. A significant equation was found (F(1,8) = 552.85, *p* < 0.001). The results of the regression indicated the velocity explained 98.7% of the variance (*R*^*2*^ = 0.987, *R*^*2*^_adj_ = 0.986) (Fig. 2.)

**Table 1:** Percent difference and descriptive statistical indicators. M refers to the mean of the measured central ablation depths at given air flow velocity

Air flow velocity	Percent difference			Collapsed indicators [μm]		
[m/s]	AB	AC	BC	M	SD	SE
4.6	0.63	0.63	0.00	63.87	0.23	0.13
4.9	0.63	1.56	0.93	64.27	0.50	0.29
5.3	0.31	1.55	1.23	64.60	0.53	0.31
5.5	0.93	2.16	1.23	64.67	0.70	0.41
5.7	0.46	1.84	1.38	65.10	0.62	0.36
6.1	0.77	2.29	1.52	65.37	0.76	0.44
6.4	0.46	1.37	0.91	65.50	0.46	0.26
6.7	0.46	1.36	0.91	65.90	0.46	0.26
7.4	0.15	0.90	1.06	66.27	0.38	0.22

## Discussion

One of the most important calibration measures is the preventive, regular maintenance and calibration of the excimer laser devices used by refractive surgery. The other important one is the analysis of optical results of artificial treatments on specific polymer plates. PMMA plates are widely used to test the ablation depth and to guarantee the refractive results of the excimer laser equipment from the first step of the design to the user-side daily calibration procedure [8–11].

Authors in a preliminary study verified that beside the optical outcomes the test treatments on PMMA are able to evaluate the corneal temperature changes during and short by after refractive treatment as well [1].

Other studies show, that shielding effect due to the insufficient air aspiration might be an important cause of asymmetrical surface after treatment with undesired refractive outcomes [16].

Others suggest that the circumstances of the treatment could have an influence on the long-term refractive results and biomechanical condition of the cornea [3, 4, 9, 17, 18].

Authors of the current study hypothesized a relationship between the shielding effect and the strength of the air aspiration. A deeper understanding of the effect of the evacuation will help to increase the predictability of the short and long-term refractive outcomes and the quality of vision.

During the study authors observed the ejection of the particles from the surface and the development of the smoke plume column without evacuation. Increasing the evacuation power this smoke plume column begins to be removed by the evacuation system. A stronger evacuation power results more evacuated part of the column and reduces the shielding effect. A linear correlation was found between the air velocity and the shielding effect.

The conclusions found are limited to PMMA and should not be directly applied to the corneal conditions. The corneal smoke plume generation and shielding differs from the PMMA [7], which is one of the limitations of this study. The examination of the repeatability with more measurements and corneal application could be the subject of future studies.

## Conclusions

Decreasing ablation effect was found due to the shielding effect of the ejected particles from the surface of PMMA plates which are widely used to calibration of excimer lasers. This effect can be caused by insufficient power of the smoke plume evacuation system. Linear correlation was found between the air flow speed and the ablation depth. It suggests an optimal ratio, and requires the regular check-ups and maintenance of the factory settings of the in-build debris removal equipment of excimer laser devices.

The debris generation, and the scattering and shielding effect depend on the ablated material. The cornea has lower particle ejection, and the dynamics of the corneal smoke plume is faster. Therefore, the obtained conclusions related to the examined PMMA material only and should not be directly extrapolated to the clinical circumstances. However the findings of our study suggest that the effect of the airflow speed to the ablation depth exist by corneal ablation as well.

The results of the study indicate the importance of the maintenance of the proper smoke plume evacuation system, which may have an influence on the short and long-term outcome of the refractive procedures performed on the cornea.

Based on the findings of this study, it is recommended to use air flow setting of the debris suction determined by the manufacturer, which should be measured and tested by every technical security check of the laser equipment.

## Abbreviations

HEPA: High-efficiency particulate arrestance
PMMA: Polymethyl methacrylate
PTK: Phototherapeutic keratectomy

## References

[1] Szekrényesi C, Sándor GL, Gyenes A, Kiss H, Filkorn T, Nagy Z (2016). Relationship between corneal surface temperature and air flow conditions during refractive laser eye surgery using three different excimer lasers. Orv Hetil.

[2] Dantas PE, Martins CL, de Souza LB, Dantas MC (2007). Do environmental factors influence excimer laser pulse fluence and efficacy?. J Refract Surg.

[3] Maldonado-Codina C, Morgan PB, Efron N (2001). Thermal consequences of photorefractive keratoectomy. Cornea.

[4] Müller B, Boeck T, Hartmann C (2004). Effect of excimer laser beam delivery and beam shaping on corneal sphericity in photorefractive keratectomy. J Cataract Refract Surg.

[5] Bor Z, Hopp B, Rácz B, Szabó G, Ratkay I, Süveges I, Füst A, Mohay J (1993). Plume emission, shock wave and surface wave formation during excimer laser ablation of the cornea. Refract Corneal Surg.

[6] Hahn DW, Ediger MN, Pettit GH (1995). Dynamics of ablation plume particles generated during excimer laser corneal ablation. Lasers Surg Med.

[7] Noack J, Tönnies R, Hohla K, Birngruber R, Vogel A (1997). Influence of ablation plume dynamics on the formation of central islands in excimer laser photorefractive keratectomy. Ophthalmology.

[8] Dorronsoro C, Siegel J, Remon L, Marcos S (2008). Suitability of Filofocon A and PMMA for experimental models in excimer laser ablation refractive surgery. Opt Express.

[9] Szekrényesi C, Réz K, Nagy ZZ (2016). Surface temperature change of PMMA plates in refractive surgery performed with two types of modern excimer lasers. New Med.

[10] Gottsch JD, Rencs EV, Cambier JL (1996). Excimer laser calibration system. J Refract Surg.

[11] Doga AV, Shpak AA, Sugrobov VA (2004). Smoothness of ablation on polymethylmethacrylate plates with four scanning excimer lasers. J Refract Surg.

[12] Naroo SA, Charman WN (2005). Surface roughness after excimer laser ablation using a PMMA model: profilometry and effects on vision. J Refract Surg.

[13] Wernli J, Schumacher S, Wuellner C (2012). Initial surface temperature of PMMA plates used for daily laser calibration to control the predictability of corneal refractive surgery. J Refract Surg.

[14] Arba-Mosquera S, Shraiki M (2010). Analysis of the PMMA and cornea temperature rise during excimer laser ablation. J Mod Opt.

[15] Zhao MH, Wu Q, Jia LL, Hu P (2015). Changes in central corneal thickness and refractive error after thin-flap laser in situ keratomileusis in Chinese eyes. BMC Ophthalmol.

[16] Dorronsoro C, Schumacher S, Pérez-Merino P, Siegel J, Mrochen M, Marcos S (2011). Effect of air-flow on the evaluation of refractive surgery ablation patterns. Opt Express.

[17] Kim JM, Kim JC, Park WC, Seo JS, Chang HR (2004). Effect of thermal preconditioning before excimer laser photoablation. J Korean Med Sci.

[18] Kymionis GD, Diakonis VF, Kounis G, Bouzoukis DI, Gkenos E, Ginis H, Yoo SH, Pallikaris IG (2008). Effect of excimer laser repetition rate on outcomes after photorefractive keratectomy. J Cataract Refract Surg.

